# Rapid Culture-Independent Detection of Fish Pathogens Using Oxford Nanopore Technologies: Case-Based Insights Across Multiple Species and Tissues

**DOI:** 10.3390/pathogens15060622

**Published:** 2026-06-10

**Authors:** Konrad Wojnarowski, Paulina Cholewińska, Dongqing Zhao, Yoshikazu Hasegawa, Daniela Denk, Dušan Palić

**Affiliations:** 1 Chair for Fish Diseases and Fisheries Biology, Ludwig-Maximilians-University Munich, 80539 Munich, Germany; p.cholewinska@lmu.de (P.C.); d.zhao@campus.lmu.de (D.Z.);; 2 Institute of Veterinary Pathology, Centre for Clinical Veterinary Medicine, Ludwig-Maximilians-University Munich, 80539 Munich, Germany; danieladenk@outlook.com

**Keywords:** aquaculture, pathogens, Oxford Nanopore Technologies, microbial diversity

## Abstract

Rapid and accurate diagnosis of infectious diseases in aquaculture is essential for preventing major economic and ecological losses. Traditional culture-based methods focus on isolation of individual pathogens, and often are burdened with extended processing times, particularly during investigations of polymicrobial infections. Application of Oxford Nanopore Technologies (ONT) sequencing offers a rapid, culture-independent workflow for the identification of bacterial and fungal pathogens directly from fish tissues. Swab and organ samples from four cases (1: *Salmo* spp.; 2: *Cyprinus carpio*; 3: *Salvelinus fontinalis*; 4: *Heniochus acuminatus*) were analyzed using ONT long-read sequencing for metagenomic screening and bioinformatic classification. The results revealed case-, species-, and tissue-specific microbial profiles, with external tissues showing higher microbial diversity and internal organs enriched in pathogenic taxa. Dominant pathogens included *Streptococcus iniae*, *Aeromonas hydrophila*, *Pseudomonas* spp., and *Saprolegnia parasitica*, alongside opportunistic zoonotic bacteria such as *Escherichia coli* and *Acinetobacter baumannii*. We demonstrate the potential for diagnostic application of ONT sequencing in investigations and detection of multi-pathogen infections, including assessments of microbial community structure changes during disease outbreaks in aquatic species. The presented workflow enables rapid, cost-effective, and comprehensive pathogen profiling, supporting early disease surveillance and improved management in aquatic veterinary practice.

## 1. Introduction

Aquaculture is the keystone industry in the world’s food supply, providing products rich in high-quality protein, healthy fats, and a variety of essential trace elements for the human body, as well as securing livelihoods and nutrition for billions of people [1]. Constraints of high-density farming include increased risk of infectious and contagious diseases caused by pathogenic microorganisms such as bacteria, viruses, fungi, and parasites, resulting in reduced productivity and increase disease burden. Farmed fishes microbiota studies are an emerging approach to assess fish health and welfare, and improve fish growth [2]. Analysis of microbial community diversity and complexity are performed with analysis of high throughput sequencing data with statistical tools to evaluate changes in the abundance of potential opportunistic and obligatory pathogens alongside with beneficial microorganisms [3,4].

Fish skin and gill mucus, and intestinal mucosa, are colonized by microbiota under normal circumstances. Microbiota such as *Proteobacteria*, *Bacteroidetes* and *Tenericutes* are commonly found in the gills of rainbow trout, while the skin contains more *Actinobacteria* and *Firmicutes* [5,6]. In fishes, the most abundant phyla found in digestive tract belong to *Proteobacteria* and *Firmicutes* [7]. While earlier thinking assumed that internal organs of healthy fish should be sterile [8], multiple recent studies confirmed that immune-related organs such as head kidneys and spleens, are also colonized by microorganisms in clinically healthy fish [9,10]. These internal organ microbiota appear to have a protective role against colonizing pathogens, as well as participate in food absorption, affecting fish reproduction and growth.

Multiple pathogens are recognized as threats to fish health worldwide. *Aeromonas salmonicida* is one example of an important bacterial pathogen, causing severe septicemia and acute mortality in susceptible hosts, and is often involved in aquaculture farms disease outbreaks, clinically observed as sluggish swimming accompanied by visceral congestion and peritonitis. The nature of the pathology and the extent of mortality are mainly related to the quality of environmental parameters and the age of the host [11]. In addition, *Aeromonas hydrophila* causes severe disease outbreaks in a wide range of freshwater fish species, often leading to high mortality rates accompanied by clinical signs such as hemorrhagic septicemia, ascites, and skin ulcerations [12]. Fish meningoencephalitis caused by *Streptococcus* spp., particularly *S. iniae*, has been recognized as a significant cause of morbidity and mortality in fish ponds. *S. iniae* causes invasive disease by colonizing the surface of fish. Infected fish show lethargy, erratic swimming, back stiffness, and die within several days [13]. Further, some of the microbiota found on fish eggs, besides environmental colonization, are also likely to be acquired from the mother, including *Saprolegnia* spp. (e.g., *Saprolegnia parasitica*), a pathogenic oomycete that causes saprolegniosis [14]. This disease is common in freshwater fish, and salmonid species such as Atlantic salmon, rainbow and brown trout are particularly susceptible to infection. Saprolegniosis usually manifests as cotton-like filamentous mycelium on fish eggs, or white and gray mycelial growth patches on the skin and fins of adult fish exposed to stressful conditions, mucosal damage, or environmental imbalance [15,16]. Fungal infections can influence the composition and function of the normal fish microbiota, increasing relative abundance of pathogens in the internal organs of diseased fish (intestines, spleen, liver, etc.) and causing a decrease in the relative abundance of beneficial genera, thereby further reducing the host’s resistance to pathogens [17].

Microbial communities play a significant role in maintaining the homeostasis of the body’s innate defenses and in prevention of pathogen colonization. Healthy fish microbiota found on mucosal or external surfaces act as a key barrier against pathogens. The stress imposed by aquaculture practices such as excessive stocking density, environmental changes, chemotherapy, or fish handling, may disrupt the microbial diversity and composition of fish skin and intestinal mucosa, thereby increasing susceptibility to opportunistic infections [18].

Early and accurate identification of pathogens is essential for diagnosis, followed by effective treatment and prevention responses to support timely interventions that can significantly reduce morbidity and mortality rates in aquaculture. Conventional diagnostic approaches initially rely on the observation of behavioral and morphological indicators to assess fish health during a clinical exam, followed by microscopic evaluation of biopsies/wet mounts, gross- and histopathology, microorganism cultures, and molecular identification methods. However, such evaluations are often confounded by extrinsic factors such as water quality (e.g., turbidity) or other technical and expertise limitations. The timeline of infection development is also important, as overt clinical manifestations typically occur only during the later stages of infection. Subclinical signs frequently remain undetectable through visual inspection during a routine health examination [19].

Culture-based methods, involving the inoculation of diseased tissue or mucus onto different media, are routinely employed for pathogen isolation. However, these methods often require multiple rounds of subculturing to obtain pure isolates and are inherently limited to the identification and characterization of a single microbial strain per assay [20]. This becomes particularly problematic in cases of polymicrobial infections, which are frequently observed during disease outbreaks in aquaculture systems. Furthermore, microbial populations may undergo genetic changes during cultivation, resulting in isolates that are not representing actual infection causatives [21]. Another obstacle is that many aquatic microorganisms are either unculturable under standard laboratory conditions, or exhibit markedly slow growth rates, thereby reducing the sensitivity and comprehensiveness of traditional diagnostics.

In recent years, advances in next-generation sequencing (NGS) have expanded the scope of fish pathogen research beyond the targeted identification of single agents, enabling broader and more detailed characterization of microbial communities directly from clinical material. Low-cost and portable sequencing platforms, such as the MinION (Oxford Nanopore Technologies, ONT), have become accessible to a wider range of users, and the overall workflow—from sample processing to taxonomic classification—can often be completed within hours to a few days. These developments create new opportunities for aquaculture diagnostic laboratories, where rapid screening is critical and polymicrobial infections are frequently encountered [22]. The long-read capability of the ONT platform supports culture-independent detection of multiple microbial taxa in a single run and facilitates profiling of microbial community structure across different tissues. As a complementary approach to conventional diagnostics, ONT-based sequencing can provide rapid and comprehensive insight into the presence of potential fish pathogens alongside the broader microbial background [19].

The identification of the microbiome of moribund fish is particularly relevant in the context of disease investigations, because these animals may harbor tissue-specific microbial signatures associated with pathogen expansion, opportunistic colonization, and disruption of the normal host-associated microbial community. Characterizing such patterns can therefore contribute not only to a better understanding of host–microbe interactions in clinically affected fish, but also to the development of rapid screening approaches for the detection of potential fish pathogens. In this way, microbiome assessment may serve as a useful complementary framework for case-based pathogen-oriented diagnostics in aquatic animal health.

Using four typical clinical cases from our diagnostic service, we demonstrate the potential for the application of the ONT sequencing in investigations and detection of multi-pathogen infections, including assessments of microbial community structure changes during disease outbreaks in aquatic species. Using ONT sequencing and corresponding bioinformatics analysis, we characterized microbial profiles across fish species and tissue types to improve understanding of host–microbe–pathogen interactions during suspected outbreaks and contribute to the development of rapid, scalable screening strategies for early detection and improved disease surveillance in aquatic animal husbandry.

## 2. Materials and Methods

### 2.1. Sample Collection and Case Descriptions

Samples were collected from four clinical cases submitted to the Chair for Fish Diseases and Fisheries Biology with a request for molecular analysis due to suspected disease outbreaks in different fish species. Samples were collected under routine diagnostic conditions by the attending veterinarian, using standard hygienic procedures intended to minimize contamination during sampling and transport.

1. “Pennant Butterflyfish” (*Heniochus acuminatus*). Multiple organ swabs were collected from clinically sick fish from a public aquarium exhibit and shipped to our laboratory under appropriate cold-chain conditions for further analysis (*n* = 1 submitted diagnostic sample).
2. “Common Carp” (*Cyprinus carpio*). Tissue samples were provided by a farmer following the observation of health issues within the stock. The samples were collected on-site and transported to our laboratory for molecular diagnostic purposes (*n* = 2 submitted diagnostic samples).
3. “Trout” (*Salmo* spp.). Swab samples were obtained from fish delivered by an aquaculture facility in response to unexplained clinical abnormalities. The fish were sampled shortly after arrival at the Chair (*n* = 2 submitted diagnostic samples).
4. “Brook Trout” (*Salvelinus fontinalis*). Samples were collected from three individual fish with clinical signs suggestive of an infectious disease problem. Skin, spleen, head kidney, liver, gill and brain swabs were taken to examine both external and internal tissues (*n* = 5).

Available case-level metadata and submitted sample types are summarized in Table 1.

### 2.2. DNA Extraction

Total DNA was extracted using the Sherlock AX Kit (A&A Biotechnology, Gdańsk, Poland), following the manufacturer’s instructions. DNA concentrations were measured using a Qubit fluorometer (Thermo Fisher Scientific, Waltham, MA, USA) with the Qubit™ 1X dsDNA HS Assay Kit (Thermo Fisher Scientific, USA), which is optimized for the quantification of low-concentration double-stranded DNA. Further QC was performed with NanoDrop 1000 Spectrophotometer (Thermo Fisher Scientific, USA).

### 2.3. Library Preparation and Sequencing

For library preparation Native barcoding V14 kit (Oxford Nanopore Technologies, Oxford, UK) with Long Fragment Buffer (LFB) in accordance with the manufacturer’s recommendations were used (Oxford Nanopore Technologies, UK). Sequencing was performed, using MinION sequencer (MIN-101B; Oxford Nanopore Technologies, Oxford, UK) with 10.4.1 Flow cell (FLO-MIN114; Oxford Nanopore Technologies, UK).

Sequences were analyzed using the Galaxy Europe web platform [23]. The assessment of read quality before and after preprocessing was performed using FastQC (v. 0.74) and NanoPlot (v.1.47.0). Trimming and filtering of reads by length and quality were conducted using Porechop (v.0.2.4). Kraken2 (v.2.1.3) and prebuilt RefSeq indexes (Prebuilt Refseq indexes: core_nt (Very large collection, inclusive of GenBank, RefSeq, TPA and PDB) (Version: 2024-09-04—Downloaded: 2025-01-04T202436Z)) were then used for classification. For levels of fungal and oomycete-associated reads, megablast was used as an additional step, including comparison against a *Saprolegnia parasitica* reference sequence (GenBank accession: KK583189.1). Host-derived reads were removed by reference-based computational filtering when a suitable host reference genome was available, in order to reduce host background prior to microbial taxonomic analysis.

For visualization of the results, the Krona chart and the R program (v4.2.3) with ggplot2, phyloseq, tidyr and vegan were used. Diversity analyses in the present study were used for descriptive comparison only and were not intended for formal inferential testing because of the limited number and heterogeneous nature of the available case-based samples.

In this study, the term “pathogen” was used for taxa previously reported in the literature as fish pathogens, opportunistic pathogens, or zoonotic bacteria of potential clinical relevance. However, because this study used PCR-free native DNA sequencing, amplification-related biases such as primer bias and differential amplification efficiency do not apply to this dataset. However, sequencing-derived relative abundance should still be interpreted cautiously, as it may be influenced by factors such as DNA extraction efficiency, host DNA background, sample composition, and taxon-specific genome characteristics.

Available sequence summary metrics for the retained ONT-derived dataset were calculated and are provided in Supplementary Table S1.

The sequencing dataset was deposited in NCBI under BioProject accession number PRJNA1471882 and is currently held under non-public/restricted status, as described in the Data Availability Statement.

## 3. Results

### 3.1. Public Aquarium

**Figure 1:**
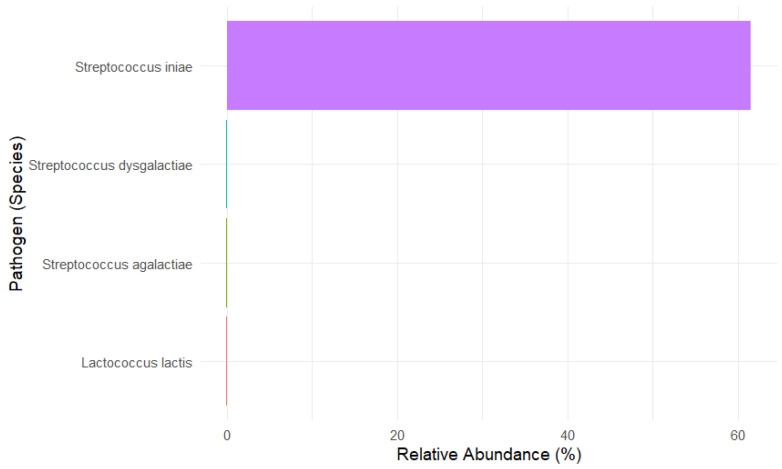
Relative abundance (%) of the four predominant fish-pathogenic bacterial species detected in swab samples collected from a clinically sick Pennant butterflyfish (*Heniochus acuminatus*) from a public aquarium—*Streptococcus iniae*, *Streptococcus dysgalactiae*, *Streptococcus agalactiae*, and *Lactococcus lactis*.

In multiple organ swabs collected from a clinically sick Pennant butterflyfish (*Heniochus acuminatus*) kept in the public aquarium, we detected that *Streptococcus iniae* was the most dominant pathogen, accounting for nearly 60% of the classified bacterial reads in the sequencing dataset. In contrast, *Streptococcus dysgalactiae*, *Streptococcus agalactiae*, and *Lactococcus lactis* were present at very low abundances, each contributing less than 1% (Figure 1).

**Figure 2:**
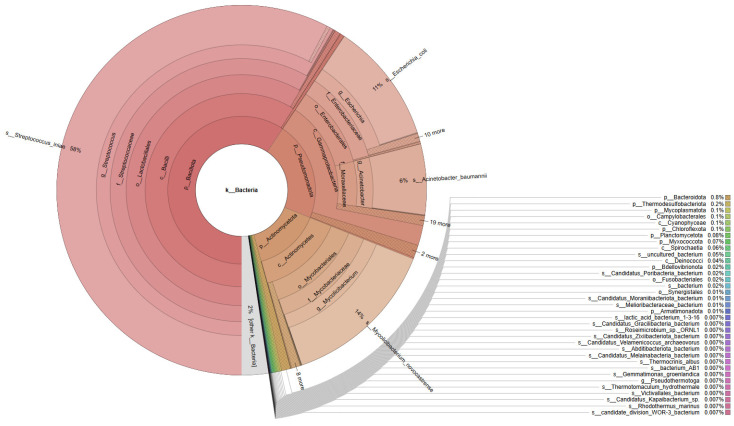
Krona chart showing the hierarchical taxonomic composition of the bacterial community in the Pennant butterflyfish (*Heniochus acuminatus*) multiple organ swab samples from a public aquarium. Segment size reflects the relative abundance of taxa across successive taxonomic levels.

The Krona chart (Figure 2) shows bacterial community was dominated by members of the phylum Bacillota, with *Streptococcus iniae* representing the most abundant species, accounting for 58% of the total bacterial population (Figure 2). Other notable taxa included *Escherichia coli* (11%), *Mycobacterium triviale* (14%), and *Acinetobacter baumannii* (6%). Additional taxa, such as members of Bacteroidota, Actinomycetota, and Pseudomonadota, were detected at relatively lower abundances (<2% each). The high prevalence of *S. iniae* highlights its dominant role within the microbial community and suggests potential pathogenic significance in the host environment.

### 3.2. Common Carp

**Figure 3:**
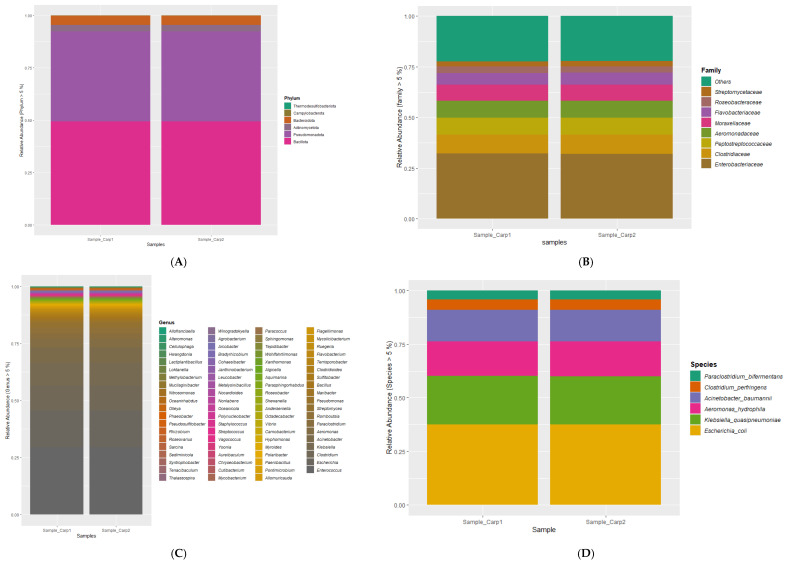
Taxonomic composition of the gut microbiota in two individual common carp (*Cyprinus carpio*) samples. Panel (**A**) shows the phylum-level distribution, while panel (**B**) presents the composition at the family level. Panel (**C**) depicts bacterial genus-level abundance, and panel (**D**) displays species-level profiles.

At the phylum level, the microbial communities in both samples are largely dominated by Bacillota and Pseudomonadota, which together account for the majority of the community composition. Other phyla detected include Actinomycetota, Bacteroidota, Campylobacterota, and Thermodesulfobacteriota, although each contributes to a smaller fraction of the total community (Figure 3A).

At the family level, diverse families were detected across both samples, including *Streptomycetaceae*, *Roseobacteraceae*, *Flavobacteriaceae*, *Moraxellaceae*, *Aeromonadaceae*, *Peptostreptococcaceae*, *Clostridiaceae* and *Enterobacteriaceae* (Figure 3B).

At the genus level, both samples exhibit similar overall taxonomic profiles (Figure 3C). Notably, genera such as *Lactiplantibacillus*, *Staphylococcus*, *Pseudoxanthobacter*, and *Vibrio* was abundant across both samples. A wide range of genera spanning phyla such as Firmicutes, Proteobacteria, Actinobacteriota, and Bacteroidota were also identified, reflecting a complex and diverse microbial community structure. In addition, fish and human pathogens such as *Aeromonas*, *Flavobacterium*, *Escherichia* and *Klebsiella* were detected. The consistency between the two samples suggests a stable microbial signature in these carp individuals under the sampled conditions.

At the species level, a diverse range of taxa was identified in both samples. These included several zoonotic pathogens, such as *Clostridium perfringens*, *Acinetobacter baumannii*, *Aeromonas hydrophila*, *Klebsiella quasipneumoniae*, and *Escherichia coli*, as well as a minor presence of *Paraclostridium bifermentans* (Figure 3D).

**Figure 4:**
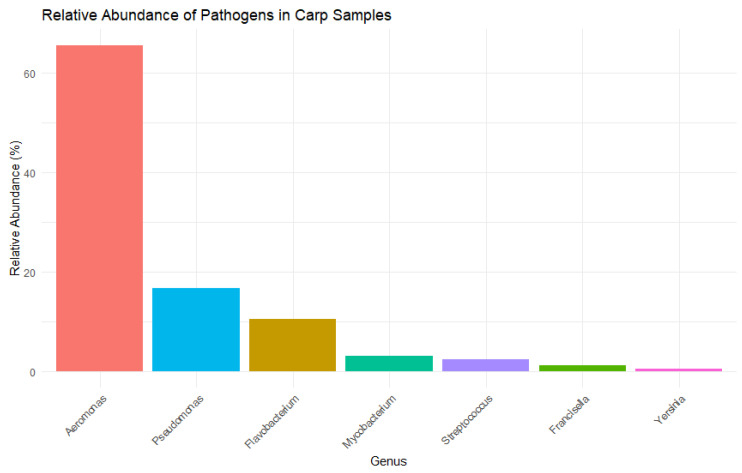
Relative abundance of selected pathogenic bacterial genera detected in the gut microbiota of two common carp (*Cyprinus carpio*) samples. The figure shows the proportional representation of genera previously associated with fish diseases, including *Aeromonas*, *Pseudomonas*, *Rhabdobacterium*, *Mycobacterium*, *Streptococcus*, *Francisella*, and *Yersinia*.

The relative abundance analysis of pathogenic genera in carp samples revealed that *Aeromonas* was the most dominant genus, accounting for over 60% of the total pathogenic community (Figure 4). This was followed by *Pseudomonas* (approximately 18%) and *Flavobacterium* (around 15%).

Other genera, including *Mycobacterium*, *Streptococcus*, *Francisella*, and *Yersinia*, were detected at comparatively lower abundances, each contributing less than 10% to the overall pathogenic composition.

### 3.3. Trout

**Figure 5:**
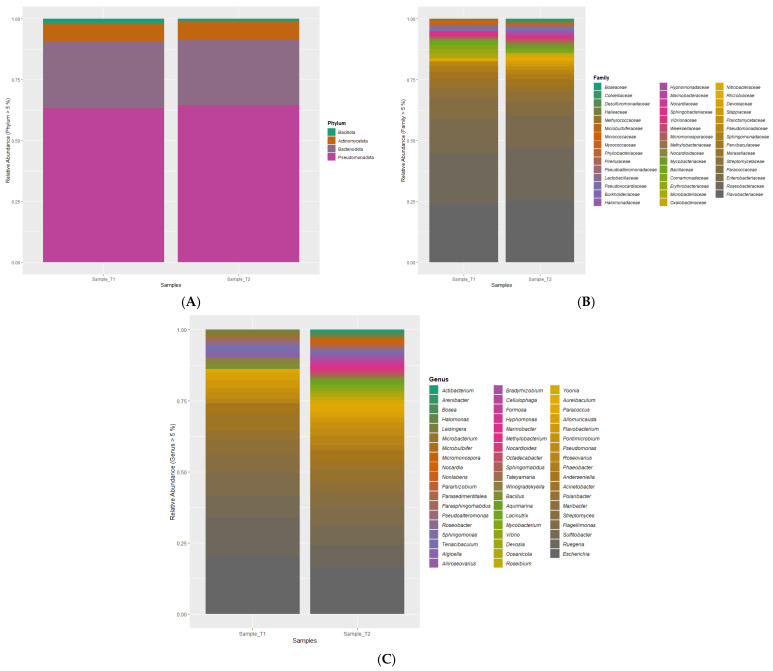
Taxonomic composition of the gut-associated microbiota in two trout (*Salmo* spp.) samples, shown at three hierarchical levels. (**A**) Relative abundance of bacterial phyla detected in each sample. (**B**) Distribution of major bacterial families shaping overall community structure. (**C**) Genus-level composition providing intermediate taxonomic resolution.

At the phylum level (Figure 5A), Pseudomonadota dominated both two samples, accounting for more than half of the total microbial community. Bacteroidota and Actinomycetota were also abundant, followed by a minor contribution from Bacillota. The overall phylum distribution patterns were highly similar between the two samples.

At the family level (Figure 5B), both Sample_T1 and Sample_T2 displayed broadly similar microbial compositions, with dominant families such as *Pseudomonadaceae*, *Burkholderiaceae*, *Halomonadaceae*, and *Roseobacteraceae*. Nonetheless, slight variations in relative abundance were noted, some families within Pseudomonadota (e.g., *Roseobacteraceae* and *Hyphomonadaceae*) showed marginally higher representation in sample_T2, while certain families associated with Actinomycetota (e.g., *Microbacteriaceae*) exhibited slight increases in sample_T1.

At the genus level (Figure 5C), the communities were highly diverse with numerous genera exceeding 5% relative abundance. Both Sample_T1 and Sample_T2 exhibited broadly similar microbial compositions, dominated by genera such as *Halomonas*, *Sphingomonas*, *Roseobacter*, and *Paracoccus*. However, obvious differences in relative abundance were observed between two samples. Certain genera affiliated with *Pseudomonadota* and *Acinetobacter* showed slightly higher proportions in Sample_T2, while others (e.g., some *Roseobacter*) appeared more abundant in Sample_T1.

**Figure 6:**
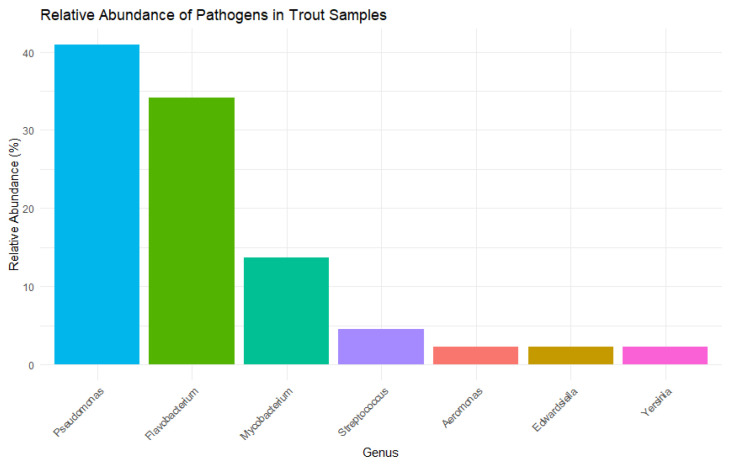
Relative abundance of pathogen-associated bacterial genera detected in two trout (*Salmo* spp.) samples—*Pseudomonas*, *Flavobacterium*, *Mycobacterium*, *Streptococcus*, *Aeromonas*, *Edwardsiella*, and *Yersinia*.

The relative abundance analysis of pathogenic genera in trout samples revealed that *Pseudomonas* was the most dominant genus, accounting for over 40% of the total pathogenic community (Figure 6). This was followed by *Flavobacterium* (approximately 35%) and *Mycobacterium* (around 13%). Other genera, including *Streptococcus*, *Aeromonas*, *Edwardsiella*, and *Yersinia*, were detected at comparatively lower abundances, each contributing less than 10% to the overall pathogenic composition.

### 3.4. Brook Trout

**Figure 7:**
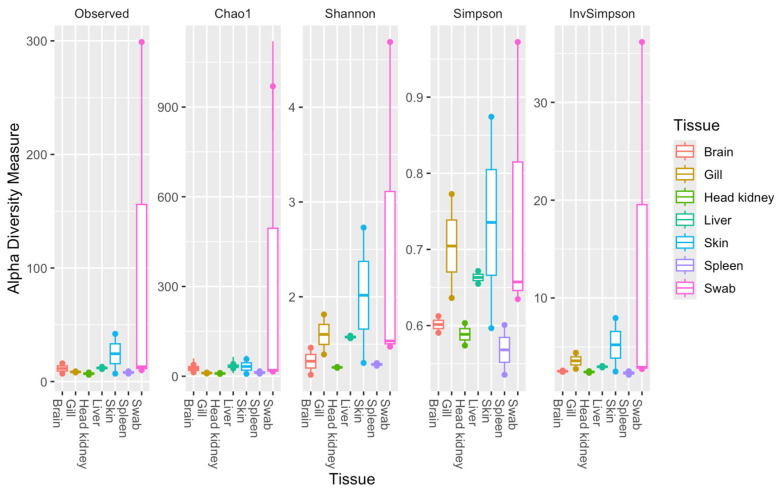
Alpha diversity across tissues of *S. fontinalis* tested samples (skin swabs, spleens, skin, livers, head kidney’s, gills and brains).

In *Salvelinus fontinalis*, alpha diversity analysis (Figure 7), assessed using the Shannon and Simpson indices, revealed clear differences in microbial diversity among the examined tissue types. Skin swab samples (including mucus) exhibited the highest diversity values across both metrics when compared to all internal organs analyzed, indicating a richer and more evenly distributed microbial community at the body surface. Gill and skin tissue samples showed intermediate diversity levels, suggesting a moderately complex microbial assemblage associated with epithelial interfaces directly exposed to the aquatic environment. In contrast, brain and head kidney tissues consistently displayed the lowest diversity values, reflecting a reduced microbial richness and/or dominance of a limited number of taxa in these internal compartments. Spleen and liver tissues were characterized by relatively low diversity, although higher than that observed in brain and head kidney samples. Overall, alpha diversity patterns suggest a gradient from highly diverse external mucosal surfaces to comparatively less diverse internal organs.

**Figure 8:**
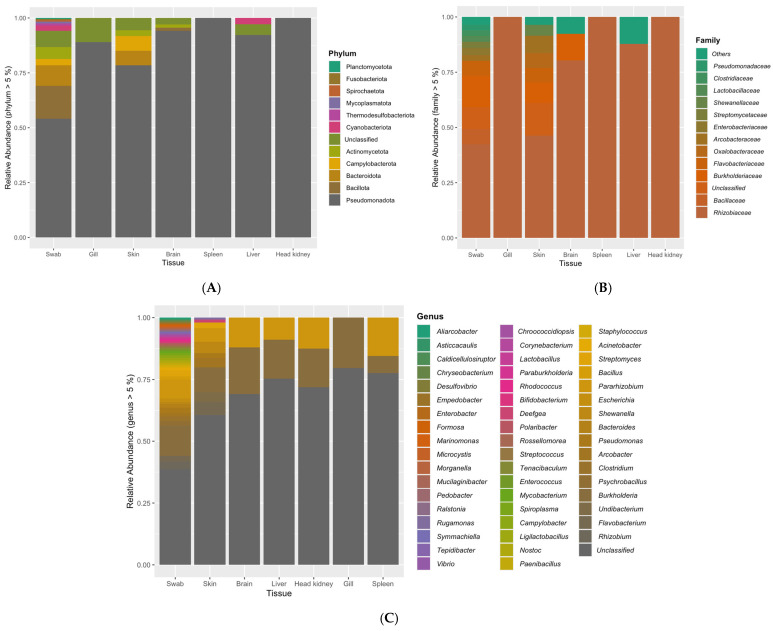
Taxonomic composition of bacterial communities across trout tissues at three hierarchical levels: phylum (**A**), family (**B**), and genus (**C**).

At the phylum level (Figure 8A), Pseudomonadota was the predominant phylum across all examined tissues; however, its relative abundance varied substantially between tissue types. Internal organs (liver, spleen, head kidney) were strongly dominated by Pseudomonadota (>80% relative abundance), whereas external surfaces (skin swab and skin tissue) displayed greater phylum-level diversity, with notable contributions from Bacillota, Bacteroidota, Campylobacterota, and Actinomycetota. Overall, phylum richness was higher in tissues directly exposed to the aquatic environment compared to internal organs.

At the family level (Figure 8B), *Bacillaceae* and *Rhizobiaceae* were consistently abundant across most tissue types and constituted the dominant families in internal organs. *Burkholderiaceae* and *Flavobacteriaceae* contributed substantially to community composition, particularly in skin and swab samples. While a set of core families was shared among tissues, external samples (swab, skin, gill) exhibited a broader family representation, including higher relative abundances of *Pseudomonadaceae* and *Shewanellaceae*. In contrast, internal organs were characterized by a stronger dominance of *Bacillaceae* and *Rhizobiaceae* and reduced overall family richness.

At the genus level (Figure 8C), tissue-specific differences became more pronounced. *Bacillus*, *Pseudomonas*, *Burkholderia*, *Flavobacterium*, and *Rhizobium* were detected across multiple tissues; however, their relative abundances differed. *Bacillus* predominated in internal organs, whereas *Pseudomonas* and *Flavobacterium* were more enriched in external samples. External tissues also harbored additional genera, including *Aliarobacter* and *Asticcacaulis*, which were absent or present at very low abundance in internal organs. Overall, genus-level richness was lower in internal organs compared to external fish surfaces.

**Figure 9:**
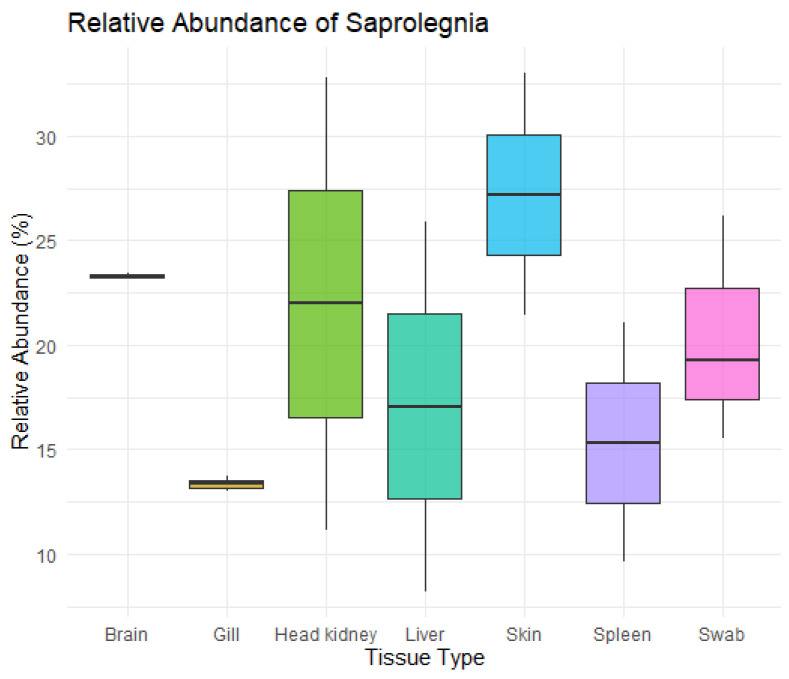
Relative abundance of Saprolegnia in brook trout (*Salvelinus fontinalis*) samples.

The relative abundance of *Saprolegnia* associated reads varied notably among different tissue types (Figure 9). The highest median abundance was observed in skin samples (approximately 27%), followed closely by head kidney (approximately 22%) and swab samples (approximately 19%). Liver and spleen tissues displayed lower median values, around 17% and 15%, respectively. Gill and brain tissues exhibited the lowest abundance. These results indicate a heterogeneous distribution of Saprolegnia associated reads across tissues, with skin and head kidney showing the highest relative abundance in the analyzed samples.

## 4. Discussion

This clinical case material–based study provides a reference for the application of high-throughput, culture-independent sequencing technologies in the diagnosis of microbial pathogens in aquaculture, including bacterial agents and oomycetes such as *Saprolegnia*, particularly in the context of polymicrobial infections. By employing Oxford Nanopore Technologies (ONT), we validated the feasibility of rapid pathogen detection directly from the clinical case-related fish biological samples. Conventional diagnostic approaches in aquatic animal health rely on obtaining pure cultures of suspected microorganisms, which are then verified using targeted immunological assays (e.g., ELISA or other antigen–antibody–based tools) and molecular methods such as qPCR.

However, this workflow is often constrained by the slow growth of many aquatic microbes and by the limited ability of antigen-based and PCR assays to detect multiple potential pathogens simultaneously [24,25]. Moreover, although advanced tools such as MALDI-TOF mass spectrometry have been recently introduced to assist with microbial identification, their high costs and infrastructure requirements limit their adoption in small-scale laboratories and veterinary practices, especially under resource-constrained or field/on-site conditions [26]. In contrast, the ONT-based method requires no bacterial isolation and enables direct DNA extraction and sequencing from different fish tissues, capturing a broader spectrum of microbial diversity. This cost-effective and portable platform can be readily implemented in veterinary practices and aquaculture laboratories with basic equipment, providing aquatic veterinarians and aquatic facility managers with a practical tool for early disease surveillance and outbreak response [27]. Although earlier ONT sequencing workflows were associated with relatively high raw read error rates, recent improvements in sequencing chemistry and base calling have substantially increased accuracy [28]. Nevertheless, ONT-derived taxonomic results should still be interpreted with appropriate caution, particularly in diagnostically relevant settings, and in the context of the bioinformatic classification strategy applied.

In addition, high levels of host-derived DNA in fish tissue samples may reduce the proportion of informative microbial reads and thereby decrease sensitivity for very low-abundance taxa. Although host-derived reads were computationally filtered in the present study, host background remains an important consideration in tissue-based metagenomic diagnostics. Moreover, the sequencing output per sample was selected as a balance between data yield, throughput, and turnaround time, consistent with the intended rapid diagnostic application. Therefore, the present workflow is intended primarily for rapid detection of clinically relevant pathogens under diagnostic conditions rather than exhaustive characterization of all rare taxa present in the sample.

Although the DNA-based ONT workflow used in this study could, in principle, detect DNA viruses or parasite-derived DNA, such detection would require additional bioinformatic analyses and interpretation specifically oriented toward these agents. These analyses were not part of the diagnostic scope of the present study. Based on the veterinary assessment of the submitted cases, the clinical presentation was not considered primarily suggestive of parasitic or viral disease; therefore, the workflow was focused on bacterial pathogens and fungal/oomycete-associated agents. RNA viruses would not be reliably detected using the present DNA-based workflow, as their detection would require RNA extraction followed by reverse transcription into cDNA and a virome-oriented analytical approach. Consequently, the absence or non-reporting of viral or parasitic agents should not be interpreted as their exclusion, and other infectious or non-infectious factors may have contributed to the observed clinical disease.

A further limitation of the present study is that extraction blanks and no-template controls were not sequenced as part of this case-based diagnostic workflow. Therefore, low-level background contamination from reagents or laboratory handling cannot be fully excluded, particularly for low-biomass material such as swab samples. This should be considered when interpreting descriptive metagenomic profiles and relative abundance patterns. To reduce the risk of overinterpretation, emphasis was placed on taxa detected at substantial relative abundance, in diagnostically relevant tissues, and in a clinical context compatible with the observed disease presentation.

Analysis of biological samples from respective example clinical cases further revealed marked differences in microbial composition and diversity across tissue types. External tissues such as skin, gills, and swabs consistently exhibited higher alpha diversity, likely due to their continuous exposure to the aquatic environment. In contrast, internal organs such as the liver, spleen, and head kidney displayed lower microbial diversity but higher relative abundances of potential pathogens, particularly in diseased individuals (Figure 7). For example, in brook trout (*Salvelinus fontinalis*) samples, the relative abundance of *Saprolegnia* was highest in the skin and head kidney tissues, may indicate these sites as key portals of entry and colonization for this opportunistic oomycete (Figure 9). Revealed distribution patterns underline the importance of sampling strategies in aquatic animal disease diagnostics, where external tissues may be more suitable for diversity profiling, whereas internal organs may more reliably reflect the distribution of clinically relevant microorganisms during disease investigations, although their detection should not by itself be taken as proof of active infection or causation [29,30].

Several pathogens previously recognized as major threats in aquaculture were also prominently detected in our report. *Streptococcus iniae*, the principal etiological agent of fish meningoencephalitis, was the dominant species in the Pennant butterflyfish from the public aquarium case, accounting for more than 60% of the microbial community. Detected prevalence is consistent with its role as a primary pathogen in the warm-water disease outbreaks and highlights the necessity of targeted surveillance in susceptible populations. Similarly, *Aeromonas hydrophila* as a hemorrhagic septicemia pathogen, was identified in the tissues of both common carp (*C. carpio*) and trout (*Salmo* spp.). Tissue distribution patterns were consistent with known Aeromoniasis pathology in fishes [31].

Zoonotic pathogens such as *Escherichia coli*, *Klebsiella pneumoniae*, and *Acinetobacter baumannii* were also detected across multiple species and tissues. Their presence underscores the public health implications of fish-borne bacterial reservoirs, especially in aquatic animal holding or production systems, where water quality management or hygienic handling may be suboptimal. The source of these bacteria cannot be conclusively determined from the present case-based sequencing data alone. More broadly, taxonomic detection alone does not necessarily distinguish between true pathogens, opportunistic colonizers, environmental organisms, and incidental contaminants, and should therefore be interpreted in conjunction with tissue origin, relative abundance, and clinical presentation. They may reflect environmental exposure, opportunistic colonization, or potential contamination introduced during sampling and laboratory processing. Rather, because the purpose of the present workflow is rapid diagnostic orientation under clinical conditions, interpretation was based on an integration of sequencing output with biological context, including clinical presentation, tissue origin, relative abundance, and the known pathogenic or zoonotic relevance of the detected taxa. These taxa are increasingly being associated with emergence of antimicrobial resistance (AMR) in clinical setting, raising concerns that aquatic environments have potential to act as reservoirs or amplifiers of AMR genes, necessitating further investigation [29,32].

## 5. Conclusions

This clinical case-based report demonstrates the feasibility and applicability of ONT as a rapid, culture-independent diagnostic tool for bacterial pathogens and oomycetes such as *Saprolegnia* in aquaculture. By extracting and sequencing the total DNA directly from different fish tissues, the method can be widely used to qualitatively identify DNA material from culturable and non-culturable pathogens, and support the process of differential diagnosis. Abundance analysis from different tissues showed higher microbial diversity in external tissues compared to internal organs; however, internal organs demonstrated higher abundance of pathogens. Aquaculture relevant pathogens, including *Streptococcus iniae*, *Aeromonas hydrophila*, *Pseudomonas* spp. and *Saprolegnia* spp., as well as zoonotic agents like *Escherichia coli* and *Acinetobacter baumannii*, have been identified, and their relative abundance indicated possible involvement in clinical disease progression and outcome in different cases. The ONT therefore shows potential as a valuable tool in fish disease diagnosis, further supporting disease control and prevention, rapid outbreak responses, and zoonotic disease risk assessment in aquatic animals.

## 6. Perspective

This report emphasizes the potential of cutting-edge sequencing methods in detecting pathogen presence and abundance in aquatic animals. ONT long-read sequencing enables rapid, culture-independent metagenomic screening, supporting simultaneous detection of multiple pathogens and providing insights into host–microbe–pathogen interactions across tissues.

## Figures and Tables

**Table 1:** Metadata are reported as available to the diagnostic laboratory at the time of sample submission. The authors were not involved in the original clinical case management, treatment decisions, or follow-up, and missing anamnestic or environmental metadata were not retrospectively inferred.

Case	Species	Water Type	Developmental Stage	Tissue/Sample Type	Clinical Information Provided	Water Temperature
Pennant Butterflyfish	*Heniochus acuminatus*	Marine/saltwater	Adult	Multiple organ swabs	Disease signs and mortality reported	25–27 °C
Common Carp	*Cyprinus carpio*	Freshwater	Adult, 3.5 kg	Tissue samples/swabs from affected skin areas	Death of the submitted fish	Not submitted with diagnostic material
Trout	*Salmo* spp.	Freshwater	Juvenile, 15–20 g	Swab samples from affected areas	Clinical signs of disease	11–14 °C
Brook Trout	*Salvelinus fontinalis*	Freshwater	Adult, 300–500 g	Skin swab, skin, gill, spleen, head kidney, liver, and brain	Clinical signs of disease	8–11 °C

## Data Availability

Sequencing data generated in this study have been deposited in NCBI under BioProject accession number PRJNA1471882 and are currently held under non-public/restricted status. The raw sequencing data are not publicly available at the time of publication due to ethical, legal, confidentiality, contractual, and consent-related restrictions associated with routine veterinary diagnostic submissions. Processed and aggregated outputs, including taxonomic summaries, sequencing output metrics, and classification results, are provided in the manuscript and Supplementary Material. The authors will seek additional authorization for broader data sharing within 12 months after publication; if such authorization is obtained, the corresponding NCBI records will be released publicly. If authorization is not obtained, the data will remain under non-public/restricted status, except where disclosure is required or justified under the applicable legal, ethical, epidemiological, confidentiality, or contractual framework.

## Supplementary Materials

The following supporting information can be downloaded at: https://www.mdpi.com/article/10.3390/pathogens15060622/s1, Table S1: Summary metrics of the retained ONT-derived sequence dataset across all analyzed clinical samples.

## Abbreviations

The following abbreviations are used in this manuscript:

ONT	Oxford Nanopore Technologies
ELISA	Enzyme-Linked Immunosorbent Assay
PCR	Polymerase Chain Reaction
qPCR	Quantitative Polymerase Chain Reaction
LFB	Long Fragment Buffer
MinION	Oxford Nanopore MinION sequencing device
FLO-MIN114	Flow Cell (type: FLO-MIN114)
FastQC	FastQC quality control software
