# Supplementary material for: Rapid Culture-Independent Detection of Fish Pathogens Using Oxford Nanopore Technologies: Case-Based Insights Across Multiple Species and Tissues
Source: Pathogens. 2026 Jun 10;15(6):622. doi: 10.3390/pathogens15060622 (PMC13305928; doi:10.3390/pathogens15060622)
Supplement: Supplementary file 1 [file pathogens-15-00622-s001.zip › pathogens-4233352-supplementary.pdf]

Supplementary Materials

Table S1. Summary metrics of the retained ONT-derived sequence dataset across all analyzed clinical samples.

Metric	Value
Number of retained sequences	32,329
Total retained sequence length	37,355,078 bp
Mean sequence length	1,155 bp
Median sequence length	706 bp
N50	1,737 bp
L50	5,651
N90	502 bp
L90	22,634
Maximum sequence length	14,329 bp
Minimum sequence length	110 bp
Sequence length standard deviation	1,168 bp
GC content	40.43%
Number of gaps	0

The aggregate retained ONT-derived sequence dataset comprised 32,329 sequences with a total retained sequence length of 37.36 Mb. Sequence lengths were relatively short, with a mean length of 1,155 bp, a median length of 706 bp, and an N50 of 1,737 bp. These values indicate a moderately fragmented dataset, which is consistent with the clinical origin of the submitted material and potential effects of sample handling, transport, DNA fragmentation, and variable host/background DNA content. Therefore, the dataset was interpreted as suitable for descriptive pathogen-oriented taxonomic screening rather than for high-resolution quantitative metagenomic profiling or technical benchmarking.
